# Supplementary material for: Uncovering de novo gene birth in yeast using deep transcriptomics
Source: Nat Commun. 2021 Jan 27;12:604. doi: 10.1038/s41467-021-20911-3 (PMC7841160; doi:10.1038/s41467-021-20911-3)
Supplement: Supplementary file 1 — Supplementary Information [file 41467_2021_20911_MOESM1_ESM.pdf]

## Supplementary Information

# Uncovering *de novo* gene birth in yeast using deep transcriptomics


## Blevins et al.


## Supplementary Tables

| Genus | Species | Sample source | Sample Strain | Reference Source | Reference Strain |
|---|---|---|---|---|---|
| *Schizosaccharomyces* | *pombe* | Verstrepen | CBS5682 | NCBI | 972h |
| *Lachancea* | *kluyveri* | Verstrepen | CBS3082 | Genolevures | CBS3082 |
| *Lachancea* | *thermotolerans* | Verstrepen | CHCC5657 | NCBI | CBS6340 |
| *Lachancea* | *waltii* | Verstrepen | CBS6430 | YGOB | Unknown |
| *Kluyveromyces* | *lactis* | Verstrepen | ATCC8585 | NCBI | NRRL Y-1140 |
| *Naumovia* | *castellii* | Verstrepen | CBS4309 | NCBI | CBS4309 |
| *Saccharomyces* | *bayanus* | SCBL | Unknown | SSS | CBS 7001 |
| *Saccharomyces* | *kudriavzevii* | Verstrepen | IFO1802 | SSS | IFO1802 |
| *Saccharomyces* | *mikatae* | Verstrepen | IFO1815 | SSS | IFO1815 |
| *Saccharomyces* | *paradoxus* | SCBL | Unknown | SSS | NRRL Y-17217 |
| *Saccharomyces* | *cerevisiae* | SCBL | S288C | SGD | S288C |

**Supplementary Table 1. Yeast species and strains used for sequencing.** Samples were obtained from the Single Cell Behavior Laboratory (SCBL) collection at the UPF and the Verstrepen Laboratory collection at the VIB. We selected the most complete reference genome and annotations available for each species at the beginning of the project; reference genome and annotation sources include the Saccharomyces genome database (SGD), SaccharomycesSensuStricto.org (SSS), the National Center for Biotechnology Information (NCBI), the Yeast Gene Order Browser (YGOB), and the Genomic Exploration of the Hemiascomycete Yeasts (Génolevures).

| Species | Assemblies | Novel | Gene annotations | Total transcripts |
|---|---|---|---|---|
| *S. cerevisiae* | 6476 | 697 | 6290 | 6987 |
| *S. paradoxus* | 6534 | 630 | 6199 | 6829 |
| *S. mikatae* | 6613 | 730 | 5997 | 6727 |
| *S. kudriavzevii* | 6441 | 722 | 5891 | 6613 |
| *S. bayanus* | 6522 | 432 | 5994 | 6426 |
| *N. castellii* | 5938 | 267 | 5870 | 6137 |
| *K. lactis* | 6056 | 929 | 5412 | 6341 |
| *L. waltii* | 6185 | 1215 | 5523 | 6738 |
| *L. thermotolerans* | 6220 | 1253 | 5498 | 6751 |
| *L. kluyveri* | 6468 | 868 | 6116 | 6984 |
| *Schizo. pombe* | 7033 | 413 | 6868 | 7281 |

**Supplementary Table 2. Number of transcripts per species.** Here are the descriptions of each row: 'Assemblies'= number of total transcripts in the *de novo* transcriptome assemblies generated with Trinity. 'Novel'= number of transcripts from the *de novo* assemblies which did not overlap any annotated features. 'Gene annotations'= number of total annotated transcripts in each species. 'Total transcripts'= number of novel transcripts plus annotated transcripts. No expression cut-off was used in this table, but the parameters used in the *de novo* assembly (Trinity) required a minimum coverage of RNA-Seq reads when evaluating each potential transfrag.

| Animal | Plant | Bacteria | Fungi (Non-ascomycota) | Protist |
|---|---|---|---|---|
| *Homo sapiens* | *Arabidopsis thaliana* | *Escherichia coli* | *Parasitella parasitica* | *Entamoeba invadens* |
| *Mus musculus* | *Zea mays* | *Paraburkholderia fungorum* | *Magnaporthe oryzae* | *Plasmodium falciparum* |
| *Gallus gallus* | *Oryza sativa* | *Bacillus subtilis* | *Zymoseptoria tritici* | *Leishmania major* |
| *Xenopus tropicalis* | *Physcomitrella patens* | *Nostoc punctiforme* | *Cryptococcus neoformans* | *Paramecium tetraurelia* |
| *Danio rerio* | *Triticum aestivum* | *Burkholderia multivorans* | *Ustilago maydis* | *Pythium irregulare* |
| *Drosophila melanogaster* | | *Actinobacteria bacterium* | *Puccinia graminis* | |
| *Caenorhabditis elegans* | | *Betaproteobacteria bacterium* | *Rhizoctonia solani* | |
| *Nematostella vectensis* | | | *Allomyces macrogynus* | |
| *Daphnia pulex* | | | *Mitosporidium daphniae* | |

**Supplementary Table 3. Outgroup species for sequence similarity searches.** The proteomes of the 35 listed species were downloaded from Ensembl and concatenated into one file. BLASTX was used to search each yeast transcript against the outgroup proteome database using an e-value cutoff of 0.05.

| | *S. paradoxus* | *S. mikatae* | *S. kudriavzevii* | *S. bayanus* |
|---|---|---|---|---|
| ***S. cerevisiae*** | *91* | *86* | *86* | *81* |
| ***S. paradoxus*** | *100* | *89* | *88* | *83* |
| ***S. mikatae*** | | *100* | *86* | *80* |
| ***S. kudriavzevii*** | | | *100* | *87* |

**Supplementary Table 4. Percentage of the genome covered by pairwise genomic alignments.** The values correspond to the percentage of total genome sequence from both species taken together in the alignment. The syntenic alignments were produced with M-GCAT. See Supplementary Table 1 for more details on the reference genomes and annotations. As expected, pairs of species which had diverged more recently had larger fractions of their genomes covered by genomic synteny blocks.

| Cut-off | conserved | genus specific | *de novo* | All |
|---|---|---|---|---|
| No minimum | 6143 | 407 | 436 | 6986 |
| > 2 TPM | 5906 | 390 | 416 | 6712 |
| > 15 TPM | 4409 | 251 | 213 | 4873 |

**Supplementary Table 5. Number of different transcripts in *S. cerevisiae* after applying expression cut-offs.** 'TPM'= transcripts per Million. 2 TPM is the lower limit of detection of our pipeline for annotated features as established by the ERCC spike-in kit, and 15 TPM reflects the lower limit for which we are able to fully reconstruct the transcript in our *de novo* assembly. We use 15 TPM as our threshold for all transcripts in the rest of our analysis as we want to ensure that we could fully assembly orthologous transcripts that may not be annotated in other species, even if they are annotated in the focal species. This decision was based on observations that there is a general correlation between the expression levels of orthologous genes.

| | ***Distant species*** | ***Schizo pombe*** | ***L. kluy L. therm L. waltii K. lactis*** | ***N. cast*** | ***S. baya*** | ***S. kudr*** | ***S. mika*** | ***S. para*** | ***S. cere-*** **specific** |
|---|---|---|---|---|---|---|---|---|---|
| | **Conserved** | | | | **Genus specific** | | **de novo** | | |
| 1.Transcriptomes | 4605 | 121 | 1361 | 48 | 194 | 90 | 88 | 114 | 363 |
| 2. Genomic synteny | 4605 | 121 | 1361 | 48 | 251 | 158 | 107 | 78 | 257 |
| 3. Paralogs | 4613 | 136 | 1346 | 48 | 247 | 160 | 106 | 73 | 257 |

**Supplementary Table 6. Determining the phylogenetic conservation of *S. cerevisiae* transcripts.** Homology detection was performed in three steps: '1. Transcriptomes'= BLAST-based searches across species at the nucleotide and protein levels. '2. Genomic synteny'= Search for expressed sequences in regions of conserved genomic synteny in the 5 *sensu stricto species*. '3. Paralogs'= First, we performed BLAST-based searches to detect paralogs of a given transcript in the same genome, then we recorded if any paralogs had more distant homologs in other species than the target transcript. 'Distant species'= If homology hits were detected by BLAST in the proteomes of the 35 outgroup species (Supplementary Table 4). In columns from '*Schizo. pombe'* through '*S. paradoxus',* the number represent transcripts with homology hits in the indicated species but not in more distant species. '*L. Kluy / L. therm / L. waltii / K. lactis'*= homology hits to at least one of the species in *Lachancea* or *K. lactis*. '*S. cere*-specific'= transcripts without homologs in any other species. See Figure 1 for complete species names and phylogenetic tree. We discarded any transcript that did not produce a hit against itself in the BLAST searches. The transcripts were classified into three groups, 'conserved', 'genus specific' or '*de novo',* depending on the depth of conservation. No expression cut-off is applied in this table.

| Protein identifier | Transcript identifier | ORF genomic location | Protein length (aa) | Protein sequence | Antisense overlapping |
|---|---|---|---|---|---|
| ORF1-1 | RUF5-1 | NC_001140.6: 212509-212704 | 64 | MNILKTIRFISQSSMTSWFL QTCYRRGICRRCYTPLGSYM IFGIVHYFCSYHIGIGTHDL HFGS | CUP1-1 |
| ORF1-2 | RUF5-2 | NC_001140.6: 214507-214702 | 64 | MNILKTIRFISQSSMTSWFL QTCYRRGICRRCYTPLGSYM IFGIVHYFCSYHIGIGTHDL HFGS | CUP1-2 |
| ORF2 | TCONS_novel_00000055 | NC_001134.8: 540804-540969 | 54 | MAANGSSMNLTGYFSPKSFN SCNSSFVKDANSILEEILTG FKDLGITIVPCLIW | ARA1 |
| ORF3 | TCONS_novel_00000461 | NC_001144.5: 947242-947398 | 51 | MQEPANFPLVSFLQDLAMKQ VSMVYQFHNIDILQVAFLAA VLLLPPIFIFV | CTR3 |
| ORF4 | TCONS_novel_00000600 | NC_001147.6:6510-6801 | 96 | MHRIPCQNVFCYNTFHEANW GYYLHLSGFHICFLDNSFNS SIVVRMAMRIYYGTHRFLRT MSIVKFKRLLGSLCGKKRIN DYQRSITFDYSHIRDI | BDS1 |
| ORF5 | TCONS_novel_00000246 | NC_001139.9:757296-757368 | 23 | MILPMSTSSGESQTGSLRSL QTA | PEX4 |

**Supplementary Table 7. Examples of proteins translated from *de novo* transcripts that overlap other genes in antisense orientation in *S. cerevisiae*.** The transcripts showed an expression level above 15 TPM in normal and/or stress conditions. Translation of the protein was assessed by the analysis of the ribosome profiling reads three nucleotide periodicity and homogeneity with RibORF; all ORFs shown had a RibORF score above 0.7 which as deemed significant. In the case of multiple ORFs with evidence of translation only the longest is shown here.

## Supplementary Figures

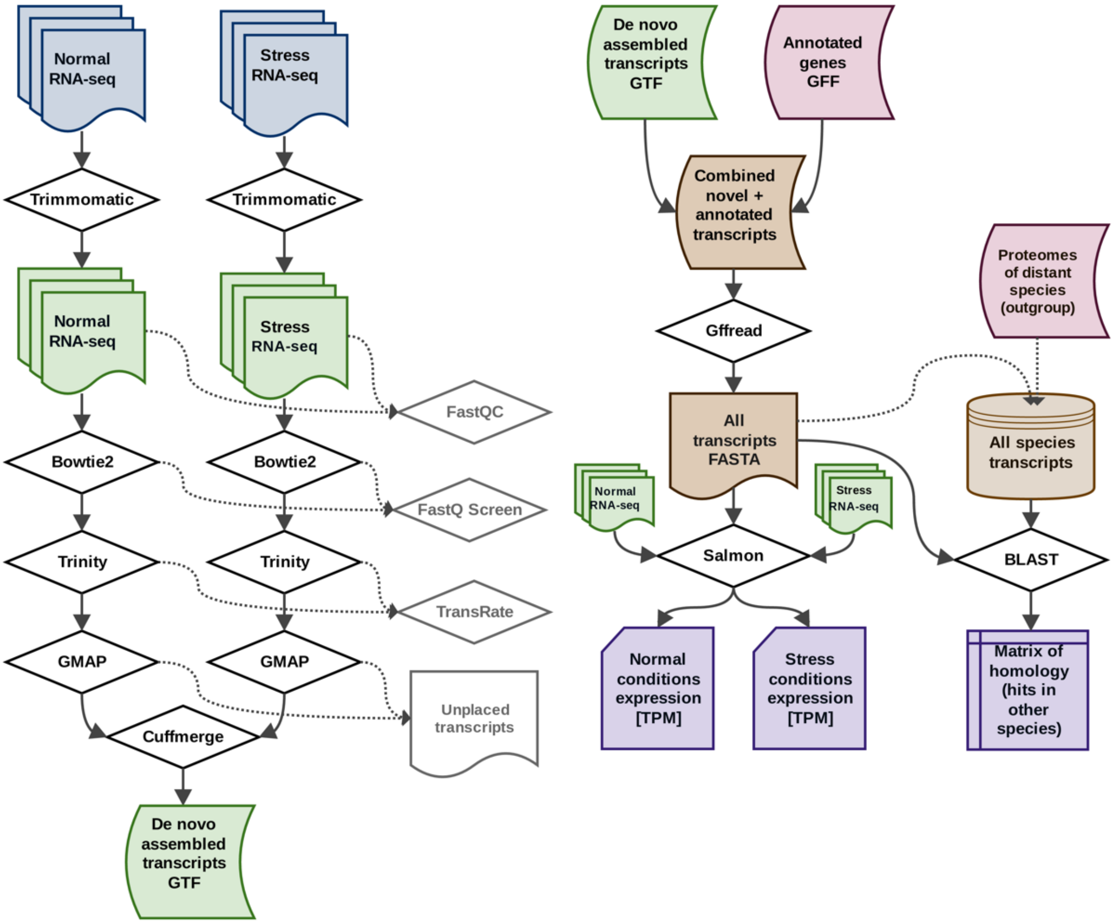


**Supplementary Figure 1. Flow chart of our RNAseq analysis pipeline.** We began our analysis with raw RNA-Seq sequencing fastq files for each of the species both conditions. Adapters and low-quality reads were removed with Trimmomatic, then FastQC was used to do a subsequent quality assessment. The high-quality reads were then mapped to the reference genome with Bowtie2. Trinity was run in reference-free mode, so the assembled transcripts it produces are lacking genomic coordinates. For this reason, we used GMAP to map where the assembled transcripts belong on the reference genome. We then used Cuffmerge to compare and combine the reference annotations with our *de novo* assembly. Nucleotide sequences were extracted for each transcript using the tool gffread from the Cufflinks suite, and BLAST databases were created for each species using the complete transcriptome (novel transcripts & annotated transcripts). Each transcript was used as a query in BLAST searches against all BLAST databases (the transcriptomes of all 11 species) as well as the proteomes of 35 distant non-Ascomycota species. Salmon was used to quantify the expression of each transcript in both conditions.

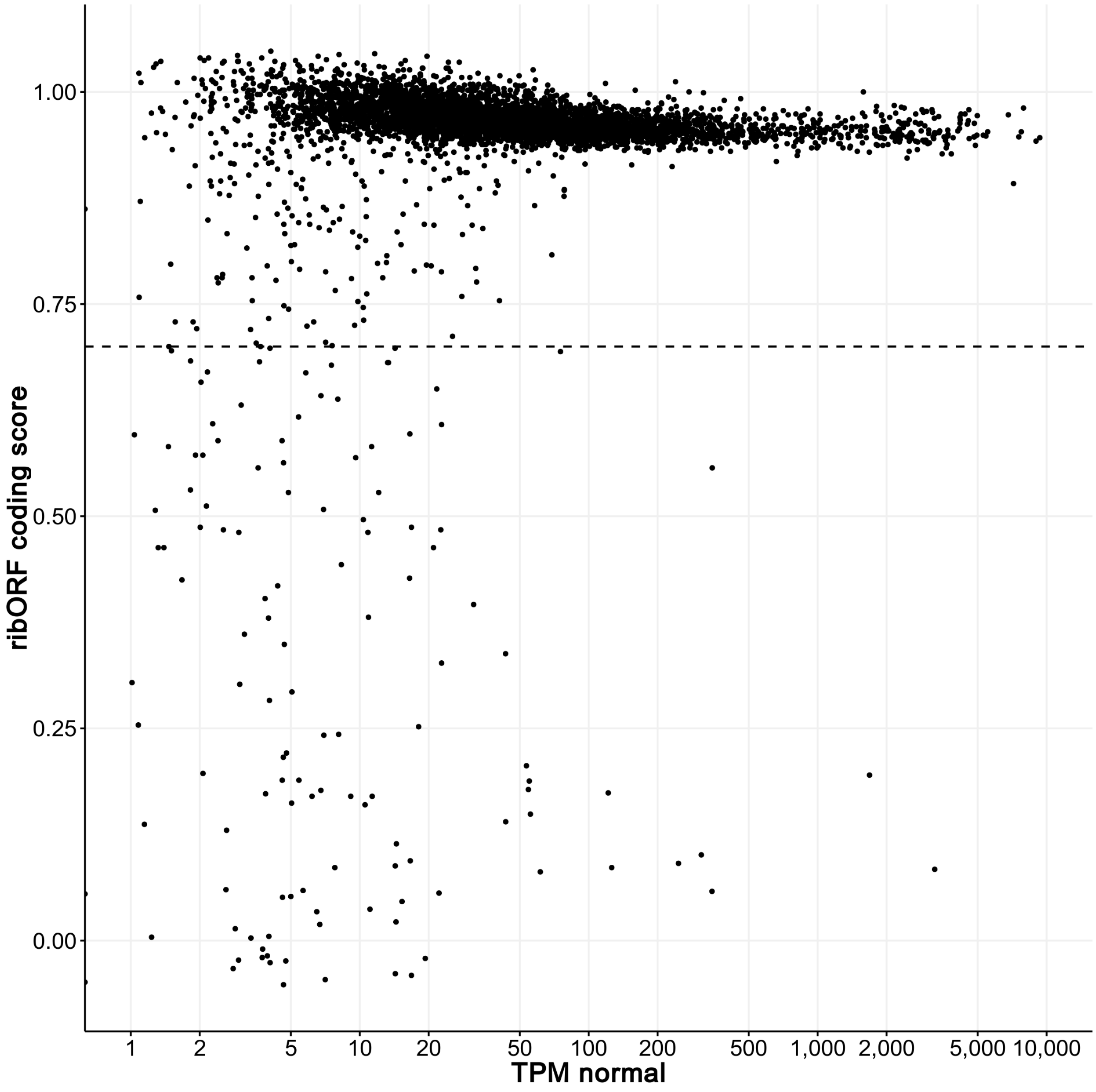


**Supplementary Figure 2. RibORF correctly classifies most verified annotated genes as coding when using our Ribo-Seq data.** We ran RibORF on a set of all verified ORFs from the *S. cerevisiae* reference annotations. Only ORFs which had at least 10 mapped ribosome profiling reads were considered in the RibORF analysis (5,068), 4932 of them had a score > 0.7 (indicated by a dotted black line). This represents a sensitivity of 97%. In the case of ORFs in transcripts expressed at TPM > 15 in at least this percentage was higher, 99% (3,820/3,851).

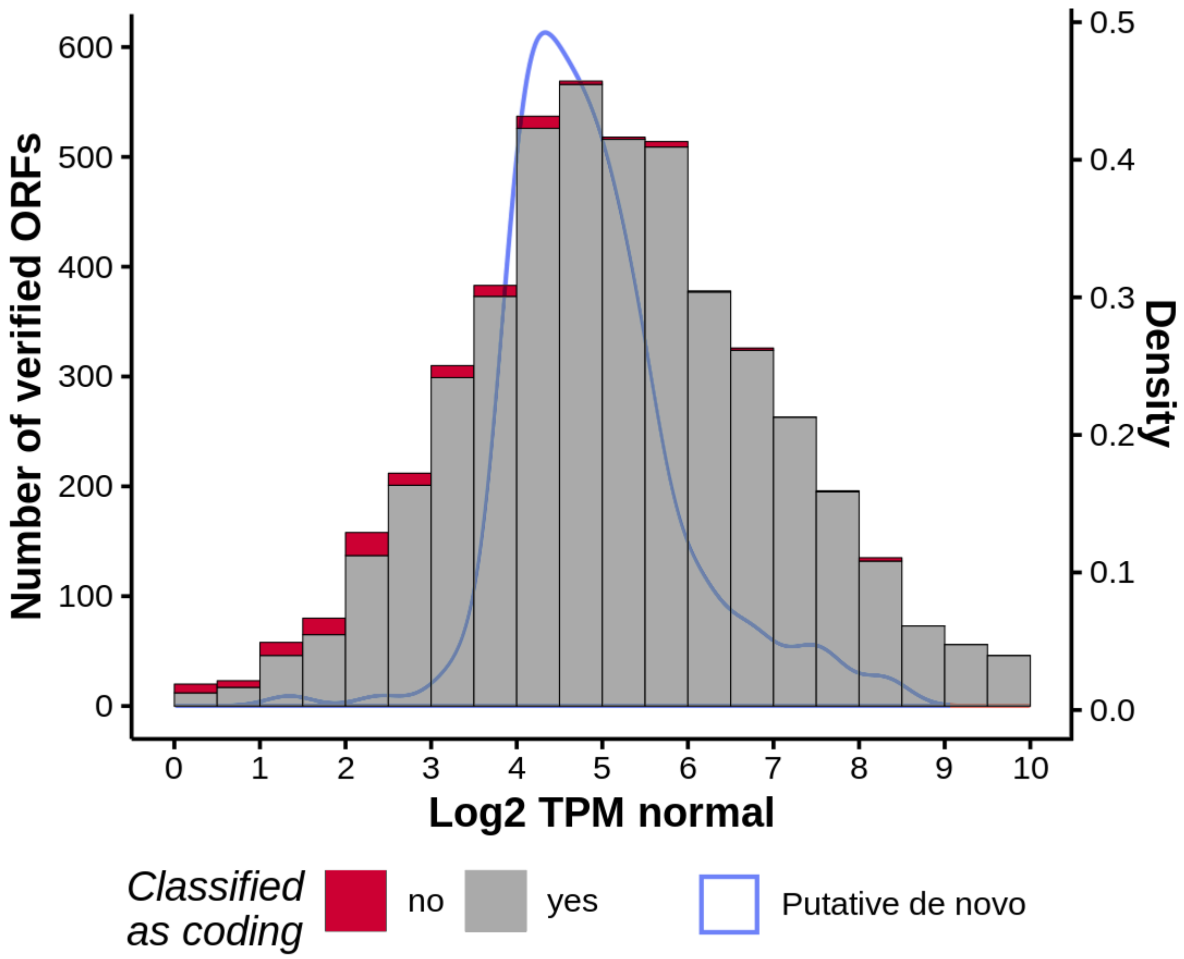


**Supplementary Figure 3. Low proportion of false negatives identified by RibORF.** Based on the accuracy of RibORF's classification of annotated protein-coding sequences i.e. verified ORFs (grey= correctly classified as translated, red= incorrectly classified as not translated) at different expression levels, we estimate that an additional 2-5% of *de novo* transcripts may be incorrectly identified as non-coding by RibORF. This is due to the fact that RibORF performs best at higher expression levels (RNA-Seq) as there are typically more ribosome protected fragments to analyze and *de novo* transcripts tend to be expressed at lower levels than annotated coding genes (light blue line). However, this 2-5% false negative rate cannot account for all *de novo* transcripts which were not found to be translated by RibORF despite their lower expression distribution.

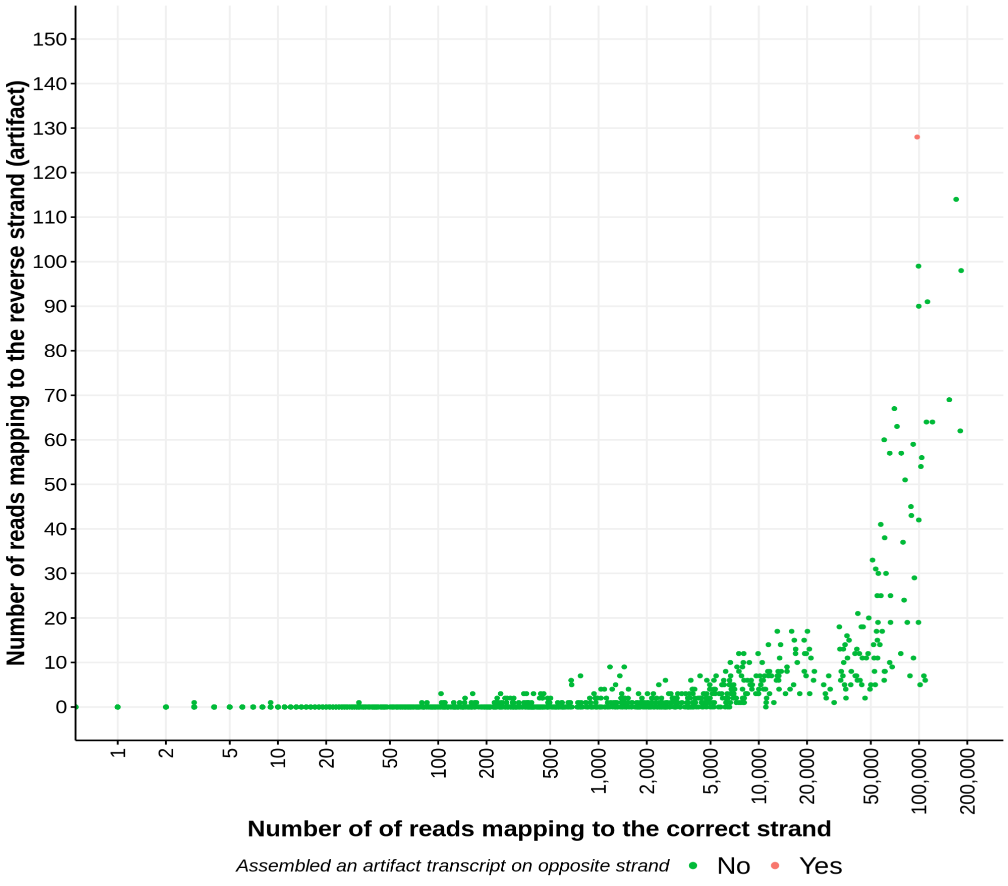


**Supplementary Figure 4. The fraction of reads that erroneously map to the antisense strand is negligible.** We included a spike-in (ERCC RNA spike-in mix 1) in each of our samples. The mix contains 92 synthetic transcripts at known concentrations. We recorded the number of reads that map to the sense and antisense strand of the ERCC RNAs. We observed that a tiny fraction of reads mapped in the opposite orientation; if there were enough of these artifacts, our pipeline could theoretically assemble a spurious novel transcript. We identified one case (shown in red) for which our pipeline assembled a spurious transcript; this occurred only for the most abundant spike-in transcript, ERCC-00130, which had a TPM over 66,000. However, this spurious transcript only appeared in one out of a possible 24 samples. In summary, we are confident that our novel transcripts which appear antisense overlapping to other transcripts are not spuriously assembled due to artifact reads which have mapped to the wrong strand.

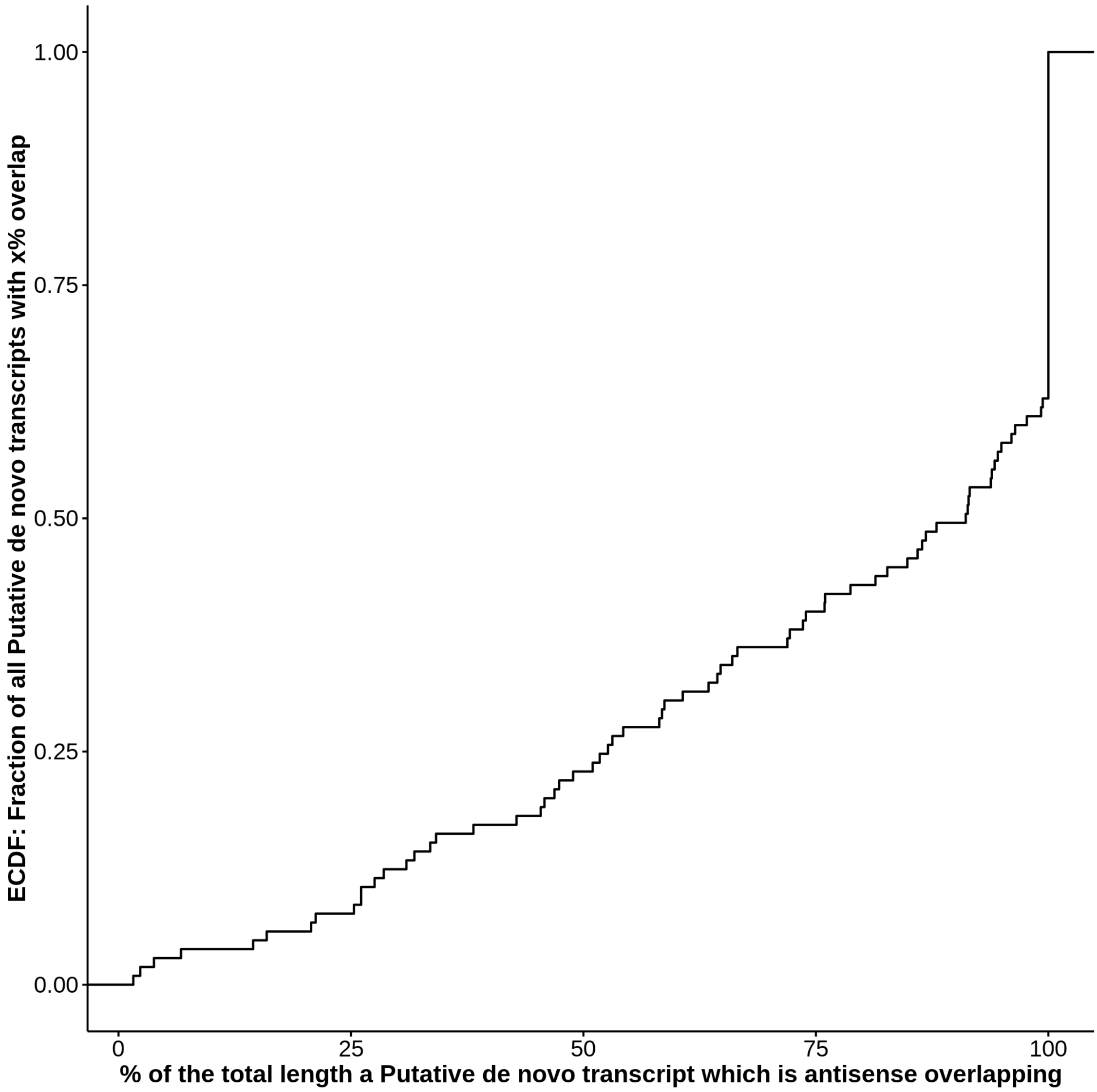


**Supplementary Figure 5. Most *de novo* transcripts have a high percentage overlap relative to their total length.** This cumulative density function shows that the vast majority of *de novo* transcripts with any antisense overlap (minimum of 1 nucleotide) have extensive overlap with the other transcript. In half of these cases, the *de novo* transcript is overlapping for ~90% or more of their total length.

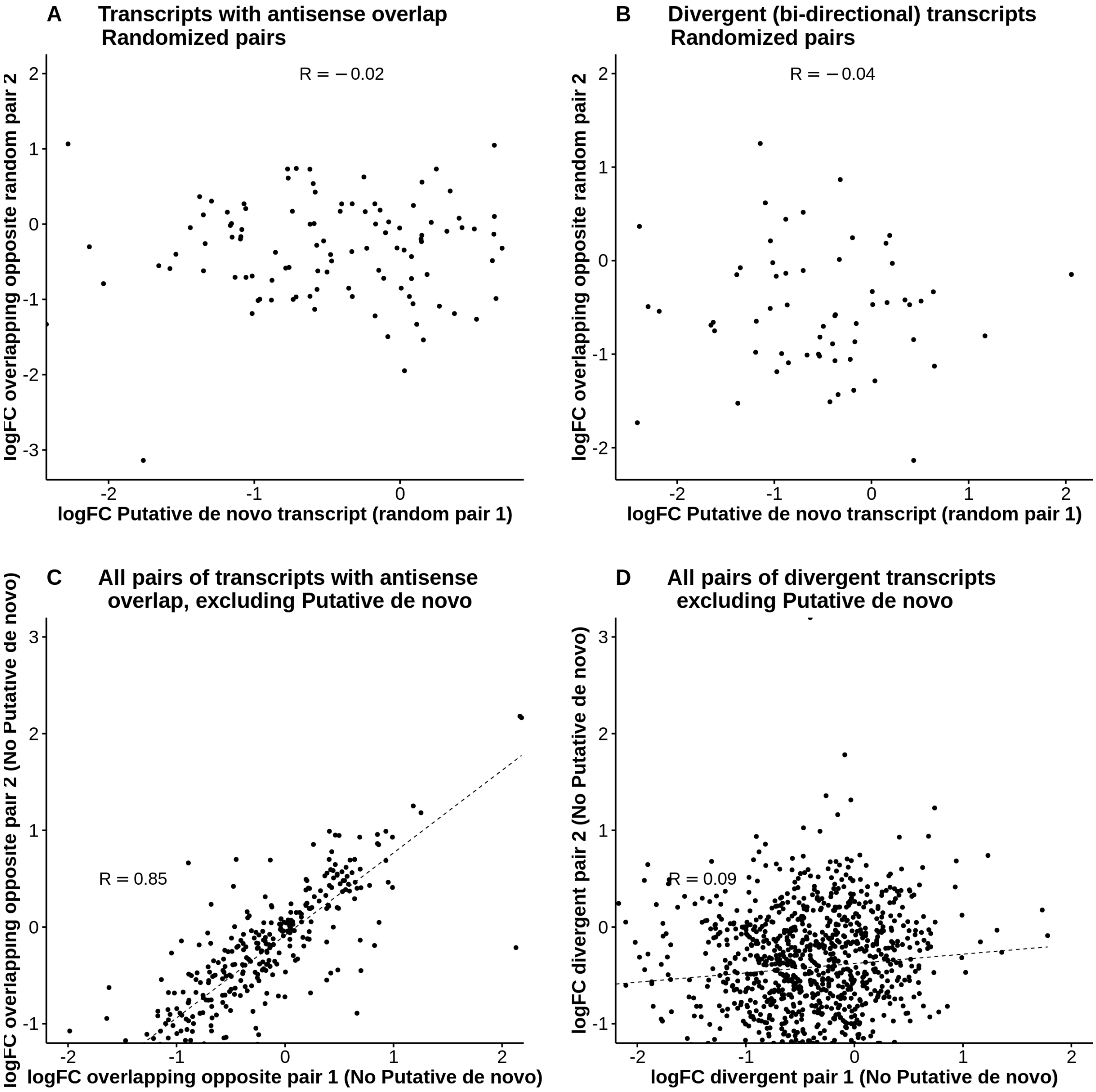


**Supplementary Figure 6. Randomized gene pairs do not show any significant correlation in expression values. A.** Log fold change (FC) of gene expression values in normal *versus* stress conditions for randomized pairs of overlapping antisense transcripts in which one of the pairs is a *de novo* transcript. Spearman's correlation -0.02 (p-value=0.8527). **B.** Log fold change (FC) of gene expression values in normal *versus* stress conditions for randomized pairs of divergent transcripts in which one of the pairs is a *de novo* transcript. The Spearman's correlation -0.04 (p-value=0.7392). **C.** Log fold change (FC) of gene expression values in normal *versus* stress conditions for pairs of overlapping antisense transcripts in which none of the pairs is a *de novo* transcript. Spearman's correlation 0.85 (p-value < $10^{-5}$). **D**. Log fold change (FC) of gene expression values in normal *versus* stress conditions for pairs of divergent transcripts in which none of the pairs is a *de novo* transcript. Spearman's correlation 0.09 (p-value < 0.006816).

## A. Mdf1 protein *S. cerevisiae*

```
>MDF1_rna537
MQYHSALYVYIYVTFTTIPYKEKPDIISICFSMLSFVFDFSVRICSRTLESFSWSLISSSAFKVVSAFSLAGSCVLASRSSVGIIVSLLLFNFSTCN
FVLFLSAVLIDLFFCTFLPTPTFLPTPFFFMLHLPIFSLLNALELLYLIIAGLHI*
```

## B. Multiple alignment genomic syntenic regions

```
                             1                                                  50
NC_001135_5_23522-23981  ~~~~~~~ATG CAGTACCATT CCGCTCTATA TGTATATAAT TACGTAACTT
Spar_3_24804-25265       ~~~~GCAGTA TAACCATTCC GCTCTCTATA TGTATATATT TACGTAACTT
Smik_3_24974-25433       ~~~~~GCAGT ATTTCATTCC CCTTTATATG TATATATTAT TACGTAACTT
Skud_3_11510-11981       GCAATATATT ATTCCTTCTC CATATGTATA TATTTATATT TACGTAACTT
Sbay_3_233283-233741     ~~~~~~~CCA GTATATTATT TCCTTCTATA TGTATATATT TACGTAACTT

                             51                                                100
NC_001135_5_23522-23981  TC.ACCACTA TTCCCTATAA GGAAAAGCCG GATATCATTT CGATCTGCTT
Spar_3_24804-25265       TC.ACCACTA TTCCCTATAA AGAAAACCCG GATATCATTT CGATCTGCTT
Smik_3_24974-25433       TC.ACCACTA TTCCTTATAA GGAAAACCCG GATATCATTT CAATCTGCTT
Skud_3_11510-11981       TTACCATCAC CCCCCTATAG GGAAAATCCG GATATCATTT CAATCTGCTC
Sbay_3_233283-233741     TC.ACCA.CC CCCCCTATAA GGAAAACCCA GATATCATCT CAATCTGCTT

                             101                                               150
NC_001135_5_23522-23981  CAGCATGCTG TCGTTTGTTT TTGATTTTTC TGTCCGAATT TGCTCTCGTA
Spar_3_24804-25265       CAGCATGTTG TCATTTGTCT TTGATTTTTC TGTCTGAATT TGTTCTCGTA
Smik_3_24974-25433       CAGCATGTTG TCATTTGTTT TTGATTTTTC CATTTGAATG TGTTCTCGTA
Skud_3_11510-11981       CAGCATGCTA TCGTTTGTCT TCGATTTTTC CGTTTGAATC TGTTCTCGCA
Sbay_3_233283-233741     CAGCATGCTG TCGTTAGTCT TTGACTTTTC TGTTTCAATA TGTTCTCGTA

                             151                                               200
NC_001135_5_23522-23981  CCTTGGAGTC CTTTTCCTGG TCCTTGATCA GCTCCTCGGC TTTCAAAGTA
Spar_3_24804-25265       CCTTGGAGTC TTTTTCCTGG TCCTTTATTA GTTCTTCAGC TTTTAGAGTA
Smik_3_24974-25433       CCTTGGAATC CTTTTCCTGG TCCTTGATCA GGTCCCCAGC TTTCAAAGTG
Skud_3_11510-11981       CCTTGCAGTC CTTTGCCTGG TCCTTAACGA GCTCTTCGGC TTTCAAAGTT
Sbay_3_233283-233741     CTTTGGAGTC CCTAGCCTGG TCCTTGACCA GCTCCTCGGC TTCCAGAGTG

                             201                                               250
NC_001135_5_23522-23981  GTCTCAGCTT TCAGTTTAGC TGGCTCTTGT GTACTTGCCT CCCGCAGCAG
Spar_3_24804-25265       GACTCAGCCT TCCTTTTGGA TGGTTCTTGC GTATTTGTCT CGCGTAACAG
Smik_3_24974-25433       GACTCAGTCT TTCTTTTAGC TGGTTCTTGT GCACCTGTCT CGCGTAGCAA
Skud_3_11510-11981       GATTTGGCCC TCTTTTTAGT CGGTTCTTGC GTGGCTGTCT CCCGTAACAG
Sbay_3_233283-233741     GACCTAGTCT TCTTCTTGTC CAGATCTTGC TTGCTTGCCT CCTTTAACAG

                             251                                               300
NC_001135_5_23522-23981  CGTCGGTATC ATTGTTTCAC TACTTTTGTT TAACTTTTCC ACTTGTAACT
Spar_3_24804-25265       CGTCGGTATC ATTGTCTCAC TACTTTTGTT TAACTTTTCC ACTTGTAACT
Smik_3_24974-25433       TGTCGGTATC ATCATCTCGC TACTTTTGTT TAACTTTTCT ACTTGTAGCT
Skud_3_11510-11981       AGTCGGTATC ATTATCTCAC TGCTTTTGTT TAGCTTTTCC ACTTGCAACT
Sbay_3_233283-233741     CGTCGGTATC ATTGTTTCGC TGCTTTTGTT TAACCTCTCC ACTTGTAACT

                             301                                               350
NC_001135_5_23522-23981  TTGTTCTCTT CCTTTCAGCG GTGCTGATCG ACCTCTTTTT TTGTACTTTC
Spar_3_24804-25265       TGGTTCTCTT CCTCTCAGCG GTGCTGATCG GCCTCTTTTT TTGTACTTTC
Smik_3_24974-25433       TGGTTCTCTT CTTCACAGCA GTGCTGATCG ACCTCCTTTT TTGCACTTTC
Skud_3_11510-11981       TGGTTCTCTT CCTCTCAGCG GTGCTGATCG GCCTCTTTTT TTGTACTTTT
Sbay_3_233283-233741     TAGTTCTTTT CATCTCAGCG GTGCTGATCG GCCTCTTTTT TTGCTGCTTC

                             351                                               400
NC_001135_5_23522-23981  TTGCCAACAC CAACATTCTT GCCCACACCT TTCTTTCTCA TGCTACACTT
Spar_3_24804-25265       TTGCCAACAG CAACATTCTT GCCCACACTC TTATTTCTCA TGCTGCTCTT
Smik_3_24974-25433       TTGCCCACAC CGACGTTCTT GCCCACACTT TTCCTTCTCA TACTGTTCTT
Skud_3_11510-11981       TTGCCAACGC CACCGTTCTT GCCCTGAGTT TTCATTCTCA AGCTGCTTTT
Sbay_3_233283-233741     TTGCCAACAC CACCGTTCTT GCCTTGAGTC TTCTTTCTCA TGCTGCTCTT

                             401                                               450
NC_001135_5_23522-23981  GCCCATTTTT TCCTTGCTAA ATGCCCTTGA GTTATTGTAC CTTATTATAG
Spar_3_24804-25265       GCCCATCTTC CT..CGTTAA ATGTCCTTGG ACTATTGTAC CTTATTATGC
Smik_3_24974-25433       GCCCATTTCT ATCTTACTAA CTGTCTTTGG C.TATTGTAC CTTATTATAC
Skud_3_11510-11981       ACCCATTTTC TTTGCGCTGA TTGTCCTTGA GCTTTTCTGT ACCTTTTAAT
Sbay_3_233283-233741     GCCCATTTTC TTCCTTCTGA ATGTCTTTGG TTTGTTGTAC CTTATTATGT

                             451                  471
NC_001135_5_23522-23981  CAGGCTTGC. ...ACATCTG A
Spar_3_24804-25265       CAGATATTGC ...ACATCTG A
Smik_3_24974-25433       CAAGTTTGCC ...ATCTGA~ ~
Skud_3_11510-11981       ATGCCAATGT TGTACATCTG A
Sbay_3_233283-233741     GATTATTGCA ...TATTTGA ~
```

## C. Computational translation in the three frames

```
>NC_001135.5:23522-23981(-) 0
MQYHSALYVYIYVTFTTIPYKEKPDIISICFSMLSFVFDFSVRICSRTLESFSWSLISSS
```

```
AFKVVSAFSLAGSCVLASRSSVGIIVSLLLFNFSTCNFVLFLSAVLIDLFFCTFLPTPTF
LPTPFFFMLHLPIFSLLNALELLYLIIAGLHI*
>NC_001135.5:23522-23981(-) 1
CSTIPLYMYIFT*LSPLFPIRKSRISFRSASACCRLFLIFLSEFALVPWSPFPGP*SAPR
LSK*SQLSV*LALVYLPPAAASVSLFHYFCLTFPLVTLFSSFQRC*STSFFVLSCQHQHS
CPHLSFSCYTCPFFPC*MPLSYCTLL*QACTS
>NC_001135.5:23522-23981(-) 2
AVPFRSICIYLRNFHHYSL*GKAGYHFDLLQHAVVCF*FFCPNLLSYLGVLFLVLDQLLG
FQSSLSFQFSWLLCTCLPQQRRYHCFTTFV*LFHL*LCSLPFSGADRPLFLYFLANTNIL
AHTFLFHATLAHFFLAKCP*VIVPYYSRLAHL
>Spar_3:24804-25265(-) 0
AV*PFRSLYVYIYVTFTTIPYKENPDIISICFSMLSFVFDFSV*ICSRTLESFSWSFISS
SAFRVDSAFLLDGSCVFVSRNSVGIIVSLLLFNFSTCNLVLFLSAVLIGLFFCTFLPTAT
FLPTLLFLMLLLPIFLVKCPWTIVPYYARYCTS
>Spar_3:24804-25265(-) 1
QYNHSALYMYIFT*LSPLFPIKKTRISFRSASACCHLSLIFLSEFVLVPWSLFPGPLLVL
QLLE*TQPSFWMVLAYLSRVTASVSLSHYFCLTFPLVTWFSSSQRC*SASFFVLSCQQQH
SCPHSYFSCCSCPSSSLNVLGLLYLIMPDIAHL
>Spar_3:24804-25265(-) 2
SITIPLSICIYLRNFHHYSL*RKPGYHFDLLQHVVICL*FFCLNLFSYLGVFFLVLY*FF
SF*SRLSLPFGWFLRICLA*QRRYHCLTTFV*LFHL*LGSLPLSGADRPLFLYFLANSNI
LAHTLISHAALAHLPR*MSLDYCTLLCQILHI*
>Smik_3:24974-25433(-) 0
AVFHSPLYVYIIT*LSPLFLIRKTRISFQSASACCHLFLIFPFECVLVPWNPFPGP*SGP
QLSKWTQSFF*LVLVHLSRVAMSVSSSRYFCLTFLLVAWFSSSQQC*STSFFALSCPHRR
SCPHFSFSYCSCPFLSY*LSLAIVPYYTKFAI*
>Smik_3:24974-25433(-) 1
QYFIPLYMYILLRNFHHYSL*GKPGYHFNLLQHVVICF*FFHLNVFSYLGILFLVLDQVP
SFQSGLSLSFSWFLCTCLA*QCRYHHLATFV*LFYL*LGSLLHSSADRPPFLHFLAHTDV
LAHTFPSHTVLAHFYLTNCLWLLYLIIPSLPS
>Smik_3:24974-25433(-) 2
SISFPFICIYYYVTFTTIPYKENPDIISICFSMLSFVFDFSI*MCSRTLESFSWSLIRSP
AFKVDSVFLLAGSCAPVSRSNVGIIISLLLFNFSTCSLVLFFTAVLIDLLFCTFLPTPTF
LPTLFLLILFLPISILLTVFGYCTLLYQVCHL
>Skud_3:11510-11981(-) 0
AIYYSFSICIYLYLRNFYHHPPIGKIRISFQSAPACYRLSSIFPFESVLAPCSPLPGP*R
ALRLSKLIWPSF*SVLAWLSPVTESVSLSHCFCLAFPLATWFSSSQRC*SASFFVLFCQR
HRSCPEFSFSSCFYPFSLR*LSLSFSVPFNMPMLYI*
>Skud_3:11510-11981(-) 1
QYIIPSPYVYIYIYVTFTITPL*GKSGYHFNLLQHAIVCLRFFRLNLFSHLAVLCLVLNE
LFGFQS*FGPLFSRFLRGCLP*QSRYHYLTAFV*LFHLQLGSLPLSGADRPLFLYFFANA
TVLALSFHSQAAFTHFLCADCP*AFLYLLICQCCTS
>Skud_3:11510-11981(-) 2
NILFLLHMYIFIFT*LLPSPPYRENPDIISICSSMLSFVFDFSV*ICSRTLQSFAWSLTS
SSAFKVDLALFLVGSCVAVSRNRVGIIISLLLFSFSTCNLVLFLSAVLIGLFFCTFLPTP
PFLP*VFILKLLLPIFFALIVLELFCTF*YANVVHL
>Sbay_3:233283-233741(-) 0
PVYYFLLYVYIYVTFTTPPIRKTQISSQSASACCR*SLTFLFQYVLVLWSP*PGP*PAPR
LPEWT*SSSCPDLACLPPLTASVSLFRCFCLTSPLVT*FFSSQRC*SASFFAASCQHHRS
CLESSFSCCSCPFSSF*MSLVCCTLLCDYCIF
>Sbay_3:233283-233741(-) 1
QYIISFYMYIFT*LSPPPL*GKPRYHLNLLQHAVVSL*LFCFNMFSYFGVPSLVLDQLLG
FQSGPSLLLVQILLACLL*QRRYHCFAAFV*PLHL*LSSFHLSGADRPLFLLLLANTTVL
ALSLLSHAALAHFLPSECLWFVVPYYVIIAYL
>Sbay_3:233283-233741(-) 2
SILFPSICIYLRNFHHPPYKENPDIISICFSMLSLVFDFSVSICSRTLESLAWSLTSSSA
SRVDLVFFLSRSCLLASFNSVGIIVSLLLFNLSTCNLVLFISAVLIGLFFCCFLPTPPFL
P*VFFLMLLLPIFFLLNVFGLLYLIM*LLHI*
```

**Supplementary Figure 7. *MDF1* ORF is not conserved beyond *S. cerevisiae*. A.** Mdf1 protein sequence, * represents a stop codon. **B.** Alignment of genomic syntenic sequences. **C.** Virtual translation of the sytenic sequences in the three possible frames. Species other than *S. cerevisiae* do not have compatible ORFs for the translation of a similar protein to Mdf1. Genomic sequences compared: NC_001135_5_23522-23981 *S. cerevisiae*, Spar_3_24804-25265 *S. paradoxus*, Smik_3_24974-25433 *S. mikatae*, Skud_3_11510-11981 *S. kudriadzevii*, Sbay_3_233283-233741 *S. bayanus*.

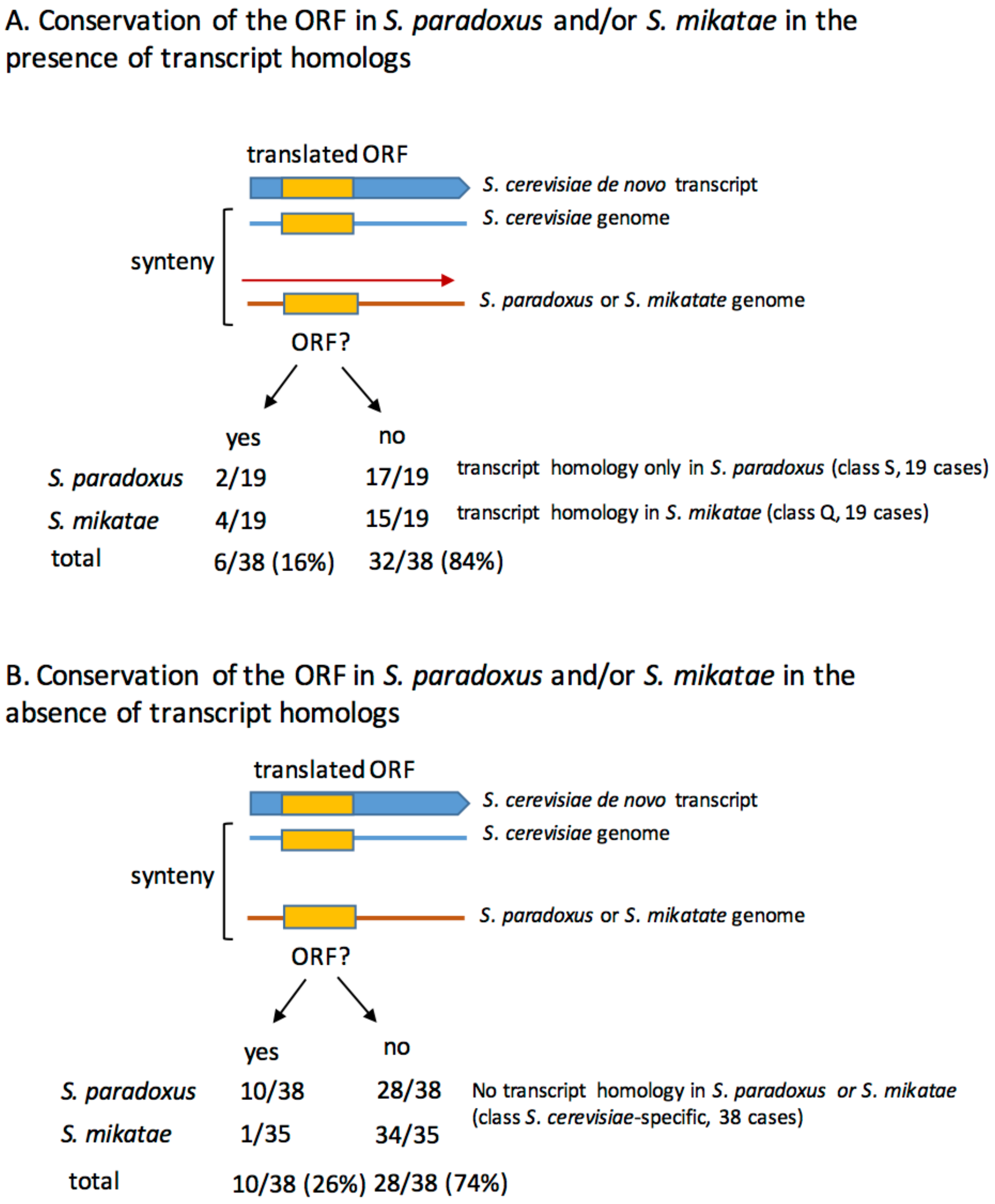


**Supplementary Figure 8. Conservation of the ORF in *S. paradoxus* or *S. mikatae,* for *S. cerevisiae de novo* transcripts with translated ORFs.** Data is for 76 transcripts with complete genomic synteny information in the other species (out of the original 99 transcripts with translated ORFs). When multiple ORFs with evidence of translation existed for a given *S. cerevisiae* transcript we focused on the longest ORF. **A.** Cases in which we detected homologs in *S. paradoxus* or *S. mikatae*. We recovered 19 cases in which there were homologs in *S. paradoxus* only and 19 cases in which there were homologs in *S. mikatae* (and optionally *S. paradoxus*) using our transcriptomics-based approach. In the majority of these cases (32/38) an equivalent ORF could not be identified in the corresponding genomic syntenic region. **B.** Cases in which we did not detect homologs in *S. paradoxus* neither *S. mikatae*. We recovered 38 cases with complete synteny in *S. paradoxus* and 35 cases with complete synteny in S. mikatae (all of which had complete synteny in *S. paradoxus*). Considering as positive the presence of an ORF in one or both species the overall result was 10 with a conserved ORF out of 38 distinct cases (26%).

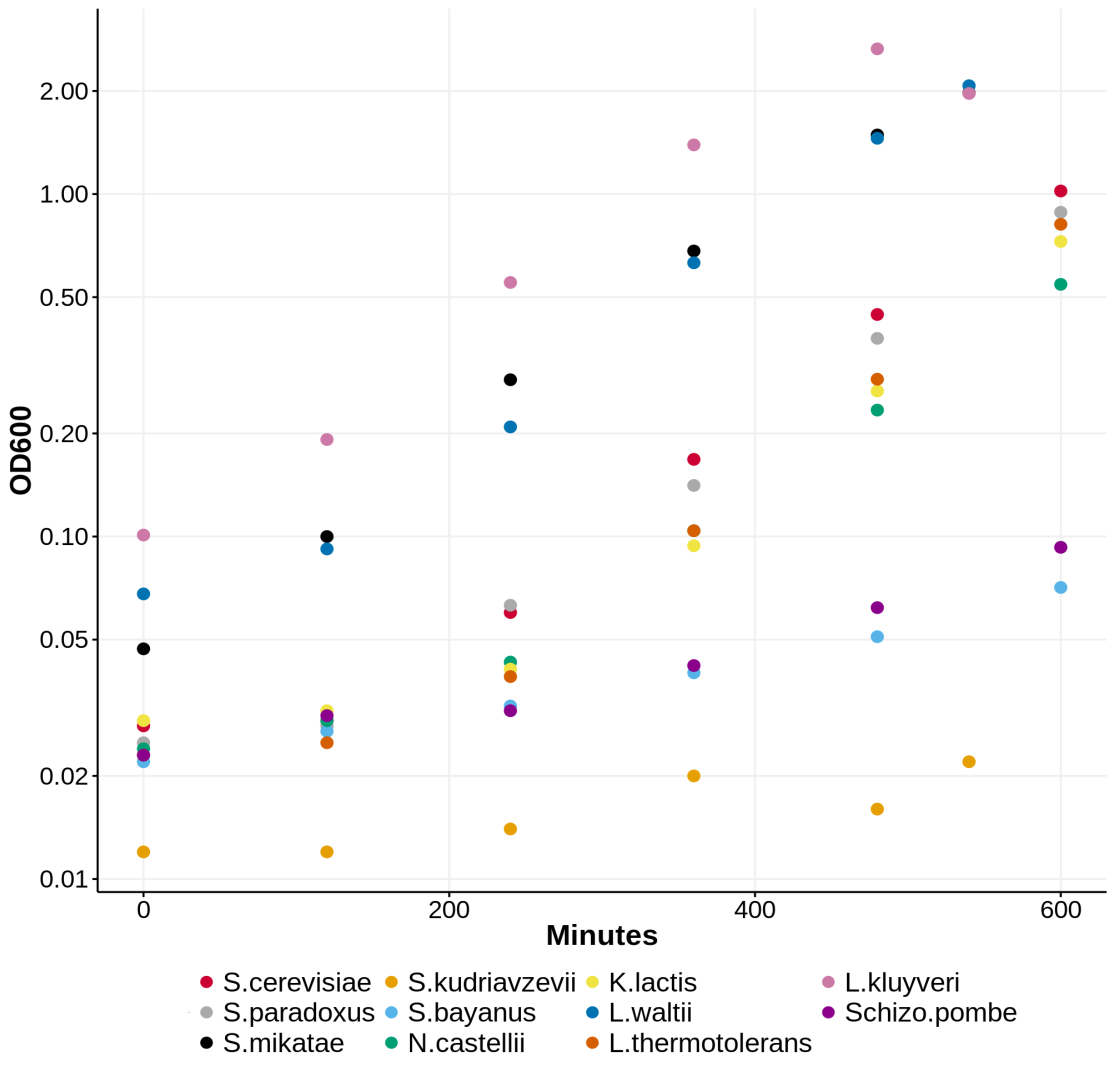


**Supplementary Figure 9. Many species of yeast grow well in the rich medium developed by Tsankov et al. 2010.** Doubling times were calculated by recording a series of measurements of the $OD_{600}$ in rich media for all 11 species. Doubling times were calculated after trimmed some time points from the beginning of the series that corresponded to lag phase.

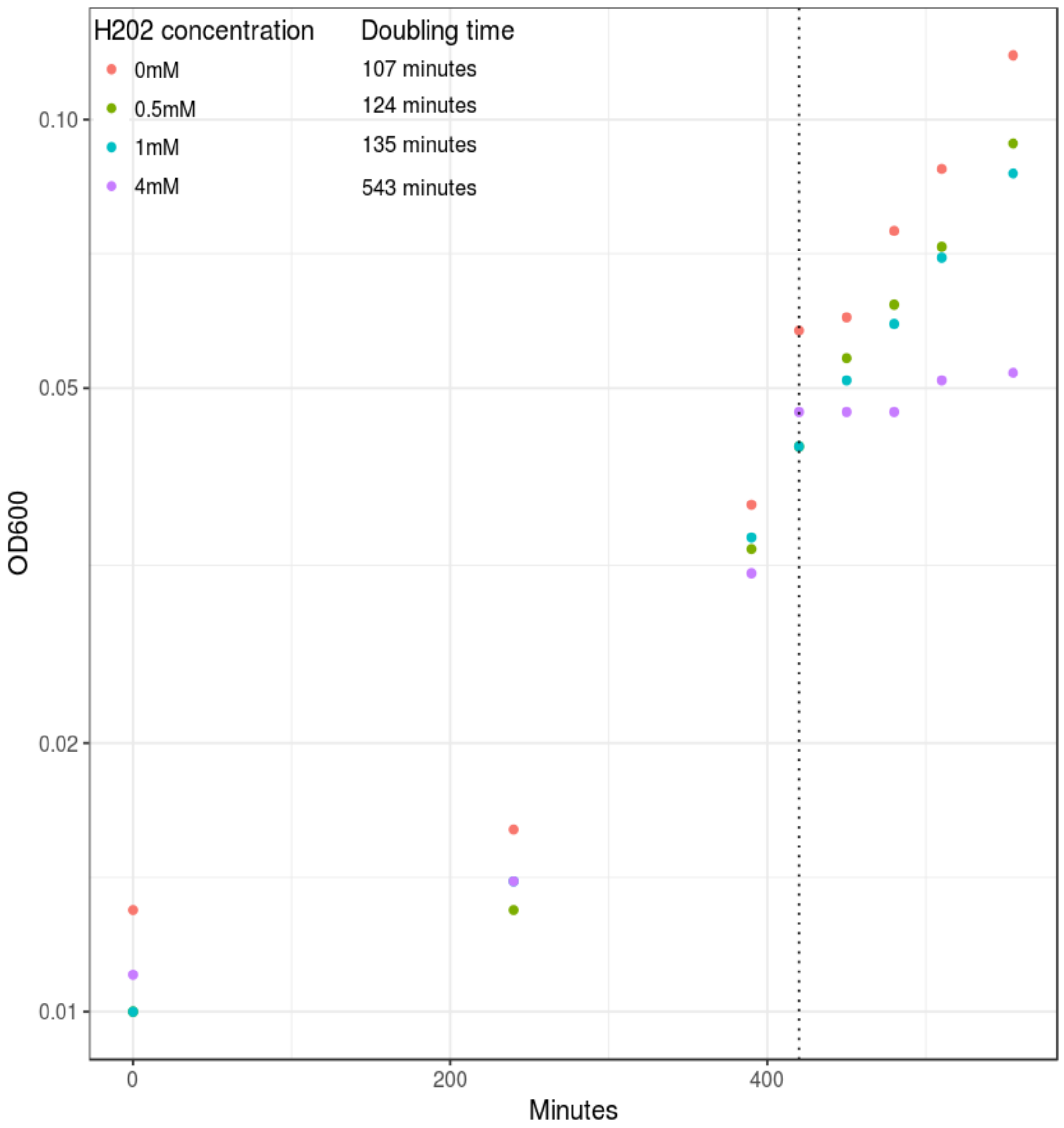


**Supplementary Figure 10. A concentration of 1.5mM $H_2O_2$ halves growth rate.** Growth rate curves were calculated for each species (*N. castellii* in this example) before and after the addition of $H_2O_2$. In this experiment the $H_2O_2$ was added to the rich medium at minute 420 which is indicated on the plot by a dashed line. The concentration of 4mM $H_2O_2$ resulted in a ~5x slower doubling time, whereas the concentration of 1mM $H_2O_2$ resulted in a ~1.26x slower doubling time. We decided to use a final concentration of 1.5mM $H_2O_2$ for an approximate 2x slower doubling time, thus halving the growth rate.

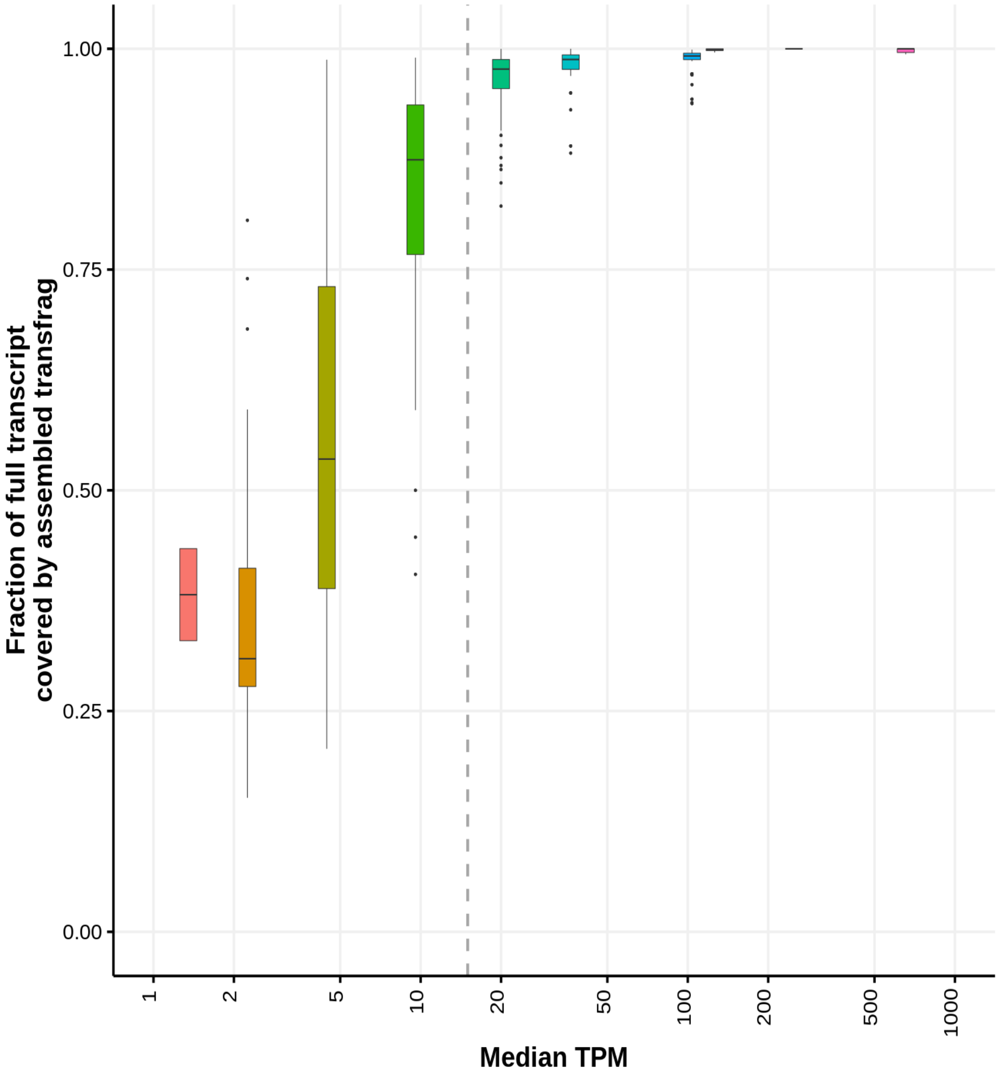


**Supplementary Figure 11. Finding the lower limit of reproducible *de novo* assembly with Trinity.** 'TPM'= transcripts per million. We attempted to assemble transcripts for each of the 92 synthetic ERCC spike-in transcripts independently in each of the 24 samples that we sequenced. As these synthetic transcripts come in different abundances, we estimated the minimum abundance necessary to consistently fully assemble the synthetic transcript with Trinity. Each colored box plot represents the subset of synthetic transcripts at different abundance levels, and each point represents the 'completeness' of the assembly for a given transcript in one sample/condition. The lower limit of our pipeline to fully and consistently reconstruct a novel transcript is approximately 15 TPM. While an incompletely assembled transcript could still be sufficient to detect homology with an ortholog in another species, we decided to err on the side of caution and only use transcripts which were likely to be fully assembled. The values of each boxplot are as follows (boxes numbered from left to right): 'box 1' min 0.33, 25% percentile 0.33, median 0.38, 75% percentile 0.43, max 0.43; 'box 2' min 0.15, 25% percentile 0.28, median 0.31, 75% percentile 0.41, max 0.59; 'box 3' min 0.21, 25% percentile 0.39, median 0.54, 75% percentile 0.73, max 0.99; 'box 4' min 0.59, 25% percentile 0.77, median 0.87, 75% percentile 0.94, max 0.99; 'box 5' min 0.91, 25% percentile 0.95, median 0.98, 75% percentile 0.99, max 1; 'box 6' min 0.97, 25% percentile 0.98, median 0.99, 75% percentile 0.99, max 1; 'box 7' min 0.99, 25% percentile 0.99, median 0.99, 75% percentile 0.99, max 1; 'box 8' min 1, 25% percentile

1, median 1, 75% percentile 1, max 1; 'box 9' min 1, 25% percentile 1, median 1, 75% percentile 1, max 1; 'box 10' min 0.99, 25% percentile 1, median 1, 75% percentile 1, max 1.

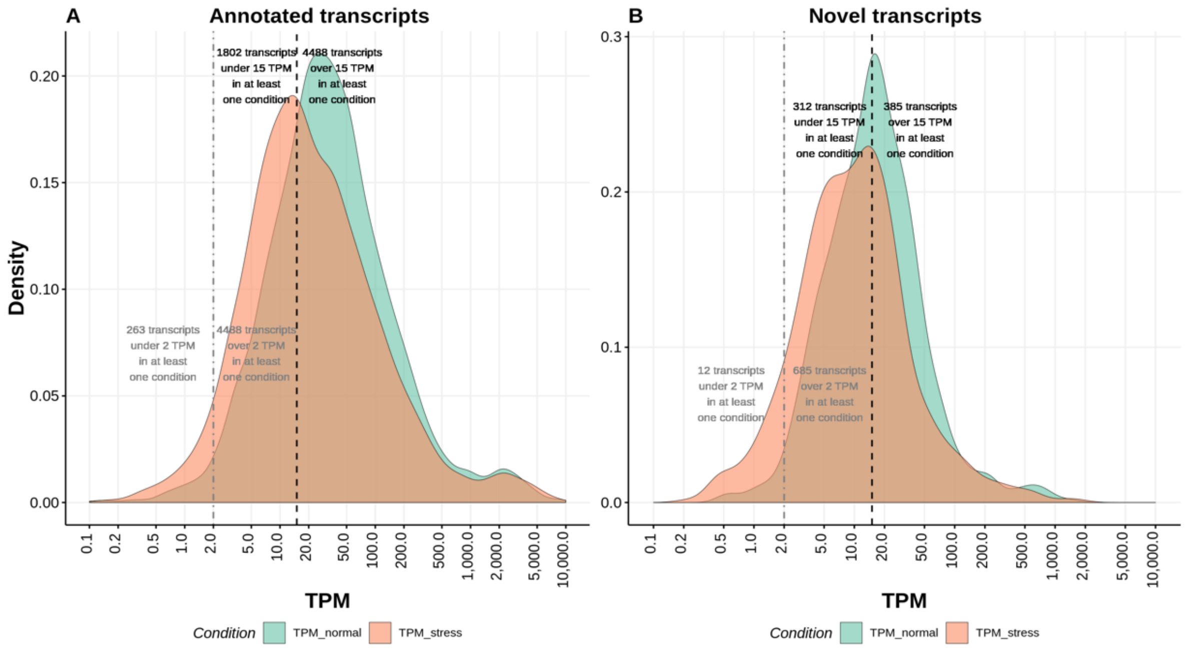


**Supplementary Figure 12. Distribution of expression levels of annotated and novel transcripts. A.** Distribution of expression of annotated transcripts in rich media (green) and oxidative stress (orange) conditions. Grey line and text indicate a TPM cutoff of >2, black line and text indicate a TPM cutoff of >15. **B.** Distribution of expression of unannotated i.e. novel transcripts in rich media (green) and oxidative stress (orange) conditions. Grey line and text indicate a TPM cutoff of 2, black line and text indicate a TPM cutoff of 15.
