# Supplementary material for: Uncovering de novo gene birth in yeast using deep transcriptomics
Source: Nat Commun. 2021 Jan 27;12:604. doi: 10.1038/s41467-021-20911-3 (PMC7841160; doi:10.1038/s41467-021-20911-3)
Supplement: Supplementary file 3 — Reporting Summary [file 41467_2021_20911_MOESM3_ESM.pdf]

nature research

Corresponding author(s): M.Mar Albà

Last updated by author(s): Dec 28, 2020

Reporting Summary

Nature Research wishes to improve the reproducibility of the work that we publish. This form provides structure for consistency and transparency in reporting. For further information on Nature Research policies, see our Editorial Policies and the Editorial Policy Checklist.

Statistics

For all statistical analyses, confirm that the following items are present in the figure legend, table legend, main text, or Methods section.

n/a	Confirmed	
☐	☒	The exact sample size (*n*) for each experimental group/condition, given as a discrete number and unit of measurement
☒	☐	A statement on whether measurements were taken from distinct samples or whether the same sample was measured repeatedly
☐	☒	The statistical test(s) used AND whether they are one- or two-sided<br>*Only common tests should be described solely by name; describe more complex techniques in the Methods section.*
☒	☐	A description of all covariates tested
☐	☒	A description of any assumptions or corrections, such as tests of normality and adjustment for multiple comparisons
☐	☒	A full description of the statistical parameters including central tendency (e.g. means) or other basic estimates (e.g. regression coefficient) AND variation (e.g. standard deviation) or associated estimates of uncertainty (e.g. confidence intervals)
☐	☒	For null hypothesis testing, the test statistic (e.g. *F*, *t*, *r*) with confidence intervals, effect sizes, degrees of freedom and *P* value noted<br>*Give P values as exact values whenever suitable.*
☒	☐	For Bayesian analysis, information on the choice of priors and Markov chain Monte Carlo settings
☒	☐	For hierarchical and complex designs, identification of the appropriate level for tests and full reporting of outcomes
☒	☐	Estimates of effect sizes (e.g. Cohen's *d*, Pearson's *r*), indicating how they were calculated

Our web collection on statistics for biologists contains articles on many of the points above.

Software and code

Policy information about availability of computer code

Data collection	No specific software was used for data collection.
Data analysis	The following publicly available software was used:<br>Trinity 2.1.0: de novo transcript assembly (Grabherr et al., 2013)<br>Transrate 1.0.3: evaluation of the quality of transcript assemblies (Smith-Unna et al., 2016)<br>GMAP version 2015-12-31: mapping the assembled transcripts back to the reference genome (Wu and Watanabe, 2005)<br>Cufflinks 2.2.0: combination of de novo assemblies with the reference transcriptome (Trapnell et al., 2012)<br>BLAST suite ncbi-blast-2.2.30+: sequence similarity searches (Altschul et al., 1997)<br>R package 3.5.1: statistical data analyses and graphs including Peptides 2.4 (R Foundation for Statistical Computing, Vienna 2016)<br>Bowtie 2-2.2.4: alignment of sequencing reads to the genome/transcriptome (Langmead and Salzberg, 2012)<br>RibORF 1.0: prediction of translated open reading frames (ORFs) in the transcripts (Ji et al., 2015)<br>CIPHER 1.0: calculation of ORF coding scores (Ruiz-Orera et al., 2018)<br>ClustalOmega 1.2.4: multiple sequence alignment (Sievers et al., 2011)<br>M-GCAT 2.0: identification of genomic syntenic regions (Treangen and Messeguer, 2006)

For manuscripts utilizing custom algorithms or software that are central to the research but not yet described in published literature, software must be made available to editors and reviewers. We strongly encourage code deposition in a community repository (e.g. GitHub). See the Nature Research guidelines for submitting code & software for further information.

Data

Policy information about availability of data

All manuscripts must include a data availability statement. This statement should provide the following information, where applicable:

- Accession codes, unique identifiers, or web links for publicly available datasets
- A list of figures that have associated raw data
- A description of any restrictions on data availability

The raw RNA sequencing data (RNA-Seq) has been uploaded to the Sequence Read Archive (SRA) under Project ID SRP187756 (https://www.ncbi.nlm.nih.gov/sra/SRP187756). Transcript assemblies can be downloaded from https//doi.org/10.6084/m9.figshare.7851521.v2. The raw ribosome profiling (Ribo-Seq) data is found under BioProject number PRJNA435567. Supplementary file contains supplementary tables and figures. Source data for figures 1 to 5 in the main manuscript is provided with the paper.

Field-specific reporting

Please select the one below that is the best fit for your research. If you are not sure, read the appropriate sections before making your selection.

[x] Life sciences [ ] Behavioural & social sciences [ ] Ecological, evolutionary & environmental sciences

For a reference copy of the document with all sections, see nature.com/documents/nr-reporting-summary-flat.pdf

Life sciences study design

All studies must disclose on these points even when the disclosure is negative.

Sample size	We used complete transcriptomics data of 11 yeast strains, no sampling was applied.
Data exclusions	No data was excluded.
Replication	NA
Randomization	NA
Blinding	NA

Reporting for specific materials, systems and methods

We require information from authors about some types of materials, experimental systems and methods used in many studies. Here, indicate whether each material, system or method listed is relevant to your study. If you are not sure if a list item applies to your research, read the appropriate section before selecting a response.

Materials & experimental systems

n/a	Involved in the study	
[x]	[ ]	Antibodies
[x]	[ ]	Eukaryotic cell lines
[x]	[ ]	Palaeontology and archaeology
[x]	[ ]	Animals and other organisms
[x]	[ ]	Human research participants
[x]	[ ]	Clinical data
[x]	[ ]	Dual use research of concern

Methods

n/a	Involved in the study	
[x]	[ ]	ChIP-seq
[x]	[ ]	Flow cytometry
[x]	[ ]	MRI-based neuroimaging
